# Normalisation of brain spectroscopy findings in Niemann–Pick disease type C patients treated with miglustat

**DOI:** 10.1007/s00415-016-8051-1

**Published:** 2016-03-16

**Authors:** Frédéric Sedel, Brigitte Chabrol, Bertrand Audoin, Elsa Kaphan, Christine Tranchant, Tomasz Burzykowski, Ayman Tourbah, Marie T. Vanier, Damien Galanaud

**Affiliations:** Department of Neurology, AP-HP, Federation of Nervous System Diseases, Salpêtrière Hospital, 47 Boulevard de l’Hôpital, 75651 Paris Cedex 13, France; Neuro-Metabolic Unit and Reference Center for Lysosomal Diseases, GRC13UPMC, Pierre and Marie Curie University, AP-HP, Salpêtrière Hospital, Paris, France; Department of Pediatrics, La Timone Hospital, CHU of Marseille, Marseille, France; Department of Neurology, Division of Clinical Neuroscience, Aix-Marseille University, CNRS, CRMBM UMR 7339, AP-HM, Timone Hospital, Marseille, France; Department of Neurology, Division of Clinical Neuroscience, CHU Timone, AP-HM, Marseille, France; Department of Neurology, CHU Hautepierre and FMTS, Strasbourg, France; International Drug Development Institute (IDDI), Louvain-la-Neuve and Hasselt University, Hasselt, Belgium; Department of Neurology, Central University Hospital, Faculté de Médecine de Reims URCA and EA 2027, Université Paris VIII, Sant-Denis, France; INSERM Unit 820, Lyon, France; Department of Neuroradiology, Pierre and Marie Curie University, Paris, France

**Keywords:** Niemann–Pick disease type C, Miglustat, Magnetic resonance spectroscopy, Substrate reduction therapy

## Abstract

Niemann–Pick disease type C (NP-C) is a fatal progressive neurolipidosis involving neuronal storage of cholesterol and gangliosides. Miglustat, an inhibitor of glycosphingolipid synthesis, has been approved to treat neurological manifestations in adults and children with NP-C. This open-label observational study in adults with confirmed NP-C evaluated the efficacy of miglustat (200 mg t.i.d.) based on composite functional disability (CFD) scores and brain proton magnetic resonance spectroscopy (H-MRS) measurement of choline (Cho)/*N*-acetyl aspartate (NAA) ratio in the centrum ovale. Overall, 16 patients were included and received miglustat for a mean period of 30.6 months: 12 continued on miglustat throughout follow up, and 4 discontinued miglustat because of adverse effects (*n* = 2) or perceived lack of efficacy (*n* = 2). In the ‘continued’ subgroup, the mean (SD) annual progression of CFD scores decreased from 0.75 (0.94) before treatment to 0.29 (1.29) during the period between miglustat initiation and last follow-up. In the discontinued subgroup, CFD progression increased from 0.48 (0.44) pre-treatment to 1.49 (1.31) at last follow up (off treatment). Mean (SD) Cho/NAA ratio [normal level 0.48 (0.076)] decreased during miglustat treatment in the continued subgroup: 0.64 (0.12) at baseline (miglustat initiation), 0.59 (0.17) at 12-month follow up, and 0.48 (0.09) at 24-month follow up. Cho/NAA ratio remained relatively stable in the discontinued subgroup: 0.57 (0.15), 0.53 (0.04) and 0.55 (0.09), respectively. In conclusion, H-MRS Cho/NAA ratio might serve as an objective, quantitative neurological marker of brain dysfunction in NP-C, allowing longitudinal analysis of the therapeutic effect of miglustat.

**Electronic supplementary material:**

The online version of this article (doi:10.1007/s00415-016-8051-1) contains supplementary material, which is available to authorized users.

## Introduction

Niemann–Pick disease type C (NP-C) is a rare lysosomal storage disorder of autosomal recessive inheritance that is characterised by progressive neurological deterioration and premature death. The incidence of NP-C has been estimated at between 1:89,000 and 1:120,000 live births [18, 33]. NP-C arises from mutations in either of two genes, *NPC1* (in 95 % of patients) and *NPC2,* which code for proteins that play roles in intracellular cholesterol and glycolipid trafficking [29]. Deficiencies in *NPC1* or *NPC2* protein products leads to the accumulation of large amounts of free cholesterol and sphingomyelin in various peripheral organs and glycosphingolipids (primarily GM2 and GM3 gangliosides) in the brain. The mechanisms through which brain gangliosides accumulate are probably multiple, and the relationship between glycolipid storage and abnormalities in cholesterol transport are still controversial [29].

While the majority of cases of NP-C reported to date relate to disease onset during infancy or childhood, increased numbers of patients with the adolescent/adult-onset form have been detected over the last decade [9, 12, 26, 30, 32]. Adult-onset NP-C is usually associated with psychiatric disorders, cognitive decline, vertical supranuclear ophthalmoplegia (VSO), cerebellar ataxia, movement disorders, gelastic cataplexy and hepatosplenomegaly [18, 31]. On average, death occurs approximately 12 years after the onset of psychiatric or neurological signs [26].

*N*-Butyl-deoxynojirimycin (miglustat) is an inhibitor of glucosylceramide synthetase that was initially approved for the treatment of Gaucher disease type I. As miglustat also inhibits the biosynthesis of all glycolipids derived from glucosylceramide, including most gangliosides, it was tested in animal models of NP-C and other neurolipidoses, and was observed to delay onset of neurological symptoms, increase survival and improve brain neuropathology [14, 27, 36]. A randomised controlled clinical trial reported stabilisation or improvement of horizontal saccade velocity, swallowing and ambulation in patients with NP-C [21]. These initial results have since been supported in numerous observational prospective or retrospective studies, small case series, and some case reports among patients with the juvenile and adult forms of the disease [4–6, 11, 15, 17, 19, 20, 22, 23, 25, 34, 35]. Clinical benefits appear less pronounced in infantile NP-C, although a degree of delay in disease progression has been reported [11, 15, 22, 23]. There is additional evidence that miglustat could stabilise or even improve some brain imaging parameters measured with positron emission tomography or diffusion tensor imaging [22, 25], as well as cerebrospinal fluid biomarkers such as β-amyloid and T-Tau [16].

Proton magnetic resonance spectroscopy (MRS) is an objective, quantitative method for measuring brain function in situ [10]. It is non-invasive and is performed routinely in most academic hospitals. The MRS choline (Cho) peak increases in several pathological circumstances associated with membrane instability including glial proliferation, myelin breakdown and abnormal lipid storage. Neurolipidoses usually cause increased choline levels in brain white matter [8, 10, 28]. In contrast, the MRS NAA (*N*-acetylaspartate) peak reflects neuronal viability, with a decrease in NAA considered as a hallmark of neuronal or axonal injury [24]. The Cho/NAA ratio integrates both choline- and NAA-related changes. Although it is not a specific parameter, it is a quantitative measure and might be useful for monitoring treatment effects in brain neurolipidoses.

In a previous publication we reported that treatment with miglustat could enable attainment of a normal Cho peak based on a follow-up study of three patients with NP-C treated for 24 months [8]. Here we investigate this finding further in a multicentre observational study in 16 adult NP-C patients treated with miglustat.

## Methods

### Patients and study design

Between March 2006 and July 2012, 16 adult patients with confirmed NP-C (based on positive filipin staining and identification of two NP-C gene mutations) were included in an observational study at three French centers: Pitié-Salpêtrière Hospital, Paris; Centre Hospitalo-Universitaire de Strasbourg; and La Timone Hospital, Marseille. Patients were included regardless of disease severity, neurological form (based on age at neurological onset), and treatment status.

Patients were evaluated every 6–12 months in line with current recommendations for the clinical management of NP-C [18]. The observation period varied from patient to patient based on the interval between the onset of neurological symptoms and last follow up. Miglustat initiation was taken as baseline for outcome assessments, with any assessments conducted before miglustat initiation assigned ‘pre-treatment’ and those conducted after miglustat initiation assigned as ‘post-baseline’. Miglustat was administered as per manufacturer’s instructions [1]. In all patients, treatment was commenced after the first appearance of neurological manifestations.

The study was coordinated by the French Committee for Evaluation of Treatments for Niemann–Pick diseases (CETNP), and was performed in accordance with relevant institutional ethical review board criteria as well as ethical standards specified in the 1964 Declaration of Helsinki and its later amendments. All patients provided written informed consent for all study procedures and data reporting.

### Functional disability assessments

At each clinical evaluation, patient functional disability was assessed using a disease-specific disability scale for NP-C that assessed four key neurological domains: ambulation, manipulation, language and swallowing [13]. A composite functional disability (CFD) score was calculated for each patient as the sum of all four individual disability domains, where scores ranged from 4 (best) to 18 (worst), as described previously [13]. For patients who commenced miglustat after onset of neurological manifestations, disability scores were calculated retrospectively between neurological onset (pre-treatment) and miglustat initiation (baseline), and prospectively throughout miglustat therapy up to last follow up (the post-baseline period). For patients who commenced miglustat at neurological onset, all disability score assessments were prospective up to last follow up.

### MRS assessments

Brain MRS was also performed at each clinic visit at Salpêtrière Hospital, Paris, France, using a 3T MR unit (General Electric, WI, USA) with a single-voxel acquisition using the PRESS sequence at long echo time (TR = 1500 ms; TE = 135 ms) in the white matter of the centrum ovale. The volume of interest was similarly located in all acquisitions and measured 40 × 16 × 20 mm (*x*, *y*, *z* axes). Spectra with a poor peak resolution were excluded from analyses. Resonances of Cho at 3.26 ppm, creatine (Cr) at 3.3 ppm, and NAA at 2.02 ppm were automatically quantified by the software provided by the manufacturer (Probe Q, General Electric Medical Systems, WI, USA). The Cho/NAA ratio was then calculated.

### Statistical analyses

Individual annual rates of progression in functional disability were calculated for each patient based on all available CFD score data obtained during pre-treatment and post-baseline observation using linear regression analysis (see supplement 1, model 1) [7]. Values were calculated for two patient subgroups: (1) patients who remained on treatment up to last follow up visit (‘continued treatment’ subgroup), and (2) patients who discontinued treatment before the last follow up visit (‘discontinued treatment’ subgroup). Pre-treatment and post-baseline annual CFD progression rates were calculated using a linear mixed effects model that included separate terms for patient subgroups and study period (‘pre-treatment’ vs ‘post-baseline’; see supplement 1, model 2). Annual rates of progression of Cho/NAA ratio were calculated similarly for individual patients for the ‘post-baseline’ period (see supplement 1, model 3).

Similar linear regression analyses were applied to assess any correlation between the post-treatment initiation annual CFD progression rate and the post-treatment initiation Cho/NAA progression rate. Raw data for MRS Cho/NAA values were calculated based on approximate time points: the actual time of measurement was within 2 months of stated time points.

All statistical tests were performed using the two-sided significance level of *α* = 0.05. All statistical analyses were conducted using SAS v. 9.3^®^.

## Results

### Patients and treatment

Individual patient data on clinical, biological and genetic characteristics are summarised in Table 1, and descriptive statistics for the overall cohort and continued and discontinued patient subgroups are provided in Table 2. Patients 1, 3–6, 9, 10 and 12–16 were followed up in the Pitié-Salpêtrière Hospital (Paris, France), patients 2, 7, 8 and 11 were followed up in La Timone Hospital (Marseille, France), and patient 14 was followed up in the Hautepierre Hospital, Strasbourg, France.

**Table 1:** Characteristics of patients and clinical evolution

Patient	G	NP-C gene mutant genotype	Age at neurological onset (years)	Age at treatment start (years)	Age at last follow-up (years)	Treatment duration (months)	Major clinical signs at treatment start	Individual disability scores and CFD at treatment start	Individual disability scores and CFD at last follow-up	Annual progression rate for ‘pre-treatment’ period^a^	Annual progression rate for ‘post-baseline’ period^b^
**Treated patients**											
1	F	p.V950M/ p.I1061T	18	22	28	70	Psy, Cog, At, Dys, VSO, Sm	A2M2L2S3 CFD = 9	A2M2L2S2 CFD = 8	0.802	−0.136
2	M	p.I1061T/ p.G538R^c^	17	20	25	61	Cog, Dys, VSO, Hea, Sm	A2M2L2S2 CFD = 8	A2M2L2S2 CFD = 8	1.397	−0.075
3	M	p.G992R/ p.N452fs	15	37	42	61	Cog, My, At, VSO, Sm	A4M4L2S2 CFD = 12	A5M4L2S3 CFD = 14	0.377	0.353
4	F	p. G992R/ p.Y1081	32	41	42	17	Cog, VSO, Sm	A2M2L2S3 CFD = 9	A2M2L2S2 CFD = 8	0.521	−0.474
5	F	p.V950M/ p.I1061T	16	22	24	22	At, Dys, VSO, Sm	A2M2L2S2 CFD = 8	A2M2L2S1 CFD = 7	−0.224	−0.560
6	M	p.Q252fs/ p.C800R	20	23	25	24	Psy, Cog, At, Dys, VSO, Sm	A2M2L2S2 CFD = 8	A2M2L2S1 CFD = 7	−0.613	−0.387
7	M	p.S954L/ p.N1156S	22	24	25	8	Psy, Cog, VSO, Sm	A1M1L2S2 CFD = 6	A1M1L2S1 CFD = 5	0.509	−1.146
8	F	p.I1061T/ p.I1061T	12	14	17	36	Not available	A3M2L1S2 CFD = 8	A3M2L2S2 CFD = 9	2.580	0.313
9	M	p.G992R+ insertion of 382 bp in intron 1	12	16	24	96	Cog, Sm, At, M, L, VSO	A2M2L2S1 CFD = 7	A2M2L2S1 CFD = 7	1.75	0
10	F	p.R389C/p.R389C	9	15	18	36	At, Dys, VSO	A3M2L2S1 CFD = 8	A3M3L2S2 CFD = 10	0.400	0.635
11	F	p.M631R/ p.R978C	15	23	23	6	At, Dys, VSO, dysphagia	A2M2L2S1 CFD = 7	A2M2L3S2 CFD = 9	NA	3.978
12	M	p.G548A/ p.I1061T	14	21	22	12	Cog, At, Dys, Sm, Psy, VSO	A3M3L2S3 CFD = 11	A3M4L2S3 CFD = 12	NA	0.984
**Discontinued patients**											
13	M	p.I1061T/p.V950M^d^	14	27	32	14	Psy, Cog, At, Dys, VSO, Sm	A4M3L2S3 CFD = 12	A5M4L3S4 CFD = 16	0.393	0.847
14	M	p. P1007A/ p. P1007A	32	34	35	6	Cog, HL, VSO, Sm	A1M1L1S1 CFD = 4	A2M2L2S1 CFD = 7	1.133	2.115
15	F	p. P1007A/ p. P1007A	17	36	38	14	Psy, Cog, At, Dys, VSO, Sm	A2M2L2S2 CFD = 8	A4M4L2S3 CFD = 13	0.205	2.977
16	M	p.C227S/ p.I841K (Dega–)	28	36	37	7	At, HL, VSO, Sm	A2M2L2S1 CFD = 7	A2M2L2S1 CFD = 7	0.203	0.039

Annual progression rates were calculated based on mixed effects linear regression model as ‘((score 1) − (score 2)/duration)’

AMLS denotes individual scores for ambulation (*A*), manipulation (*M*), language (*L*), swallowing (*S*), *At* ataxia, *bp* base pairs, *CFD score* composite score derived from individual functional disability scores, *Cog* cognitive disorder (excluding psychiatric symptoms), *Dys* dystonia, *F* female, *G* gender, *HL* hearing loss, *M* male, *My* myoclonus, *Psy* psychiatric signs, *SMA* spinal muscular atrophy, *Sm* splenomegaly

^a^For rates before treatment ‘score 1’ is CFD score at treatment initiation, ‘score 2’ is CFD score at neurological onset (which is set to 4) and ‘duration’ is the number of years between neurological onset and treatment initiation

^b^For rates after treatment, ‘score 1’ is CFD score at last follow-up, ‘score 2’ is CFD score at treatment initiation, and ‘duration’ is the number of years between treatment initiation and last follow-up

^c^Patient details published previously in Sevin et al. [26], Brain 130:120–133

^d^Patient details published previously in Millat et al. 2001, Am J Hum Genet 68:1373–1385

**Table 2:** Clinical characteristics for overall patient cohort and continued and discontinued treatment subgroups

	Whole cohort (*N* = 16)	Continued treatment subgroup (*N* = 12)	Discontinued treatment subgroup (*N* = 4)
Age at neurological onset (years)			
Mean (SD)	18.3 (6.9)	16.8 (6.0)	22.8 (8.6)
Median (range)	16.5 (9–32)	15.5 (9–32)	22.5 (14–32)
Age at treatment start (years)			
Mean (SD)	25.7 (8.5)	23.2 (8.1)	33.3 (4.3)
Median (range)	23.0 (14–41)	22.0 (14–41)	35.0 (27–36)
Treatment duration (months)			
Mean (SD)	30.6 (27.3)	37.4 (28.5)	10.3 (4.4)
Median (range)	19.5 (6–96)	30.0 (6–70)	10.5 (6–14)
Age at last follow-up (years)			
Mean (SD)	28.6 (8.1)	26.3 (8.0)	35.5 (2.7)
Median (range)	25.0 (17–42)	24.5 (17–42)	36.0 (35–38)
Total clinical observation period (years)^a^			
Mean (SD)	8.7 (7.8)	7.3 (7.2)	13.4 (9.3)
Median (range)	6.1 (1–25)	5.1 (1–25)	15.1 (3–22)

^a^Based on number of patients with available CFD data (*n* = 13 overall, *n* = 10 continued treatment patients, and *n* = 4 discontinued treatment patients)

There were nine males and seven females, overall. The median (range) age at neurological symptom onset in the whole cohort was 16.5 (9–32) years, and the median (range) age at treatment start was 23.0 (14–41) years. Both median age at neurological onset and median age at treatment start tended to be higher in patients who discontinued miglustat during the observation period, than in those who continued on miglustat.

In the whole study cohort, patients were treated with miglustat for a mean (SD) of 30.6 (27.3) months; median (range) 19.5 (6–96) months. Miglustat was well tolerated in the 12 patients in whom therapy was continued throughout follow up (the ‘continued’ subgroup). Patients 13–14, 15 and 16 discontinued miglustat therapy before the last follow-up visit. These patients (the ‘discontinued’ subgroup) spent a mean (SD) period of 16.6 (14.6) months off treatment between miglustat initiation and last follow up. Treatment was stopped after 14 months in patients 13 and 15 due to continuous symptom progression and severe dementia. Patient 14 stopped treatment after 6 months due to lack of motivation following weight loss and tremor. Patient 16 stopped treatment after 1 month because of severe depression and attempted suicide. He then re-started treatment when his depressive thoughts resolved, but a later recurrence of severe depression and autoagressive behaviour led to definitive treatment discontinuation after an overall period of 5 months on miglustat.

### Functional disability

The mean (SD; range) total period for CFD assessments (i.e., the sum of pre-miglustat initiation retrospective assessments and assessments conducted during follow-up after miglustat initiation) was 8.7 (7.8, 1–25) years. The mean (SD; range) observation periods between miglustat initiation and last follow up in the continued subgroup (*n* = 12) and the discontinued subgroup (*n* = 4) were 7.3 (7.2, 1–25) years and 13.4 (9.3, 3–22) years, respectively (Table 2).

**Fig. 1:**
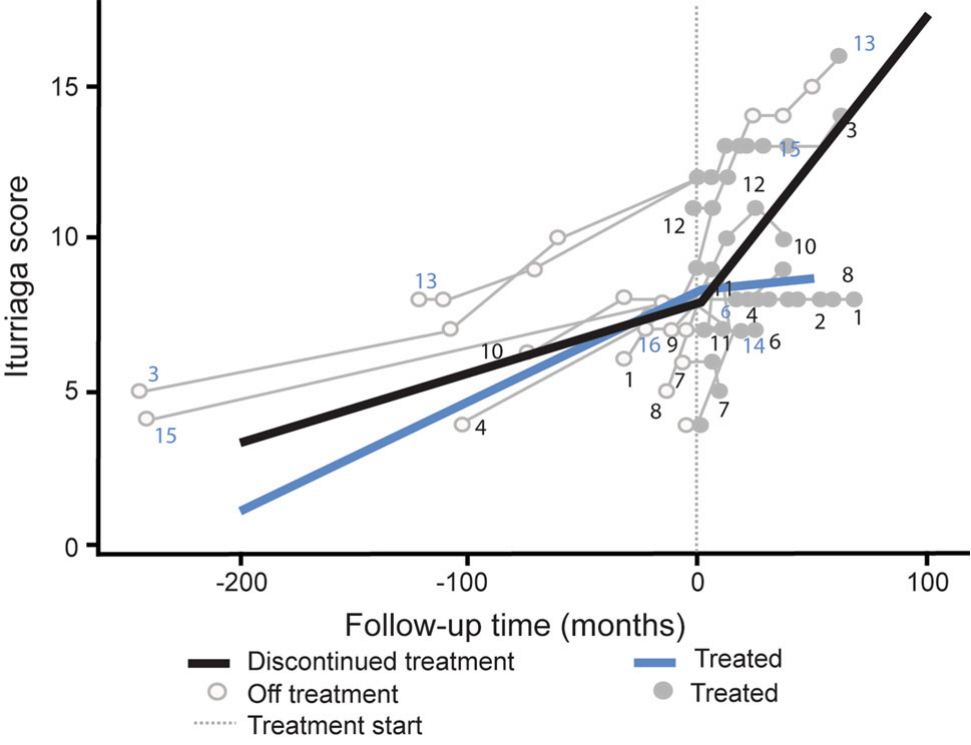
CFD score progression over time. Data based on total observation period across all patients. Zero time point on x-axis represents start of miglustat treatment; *thick coloured lines* represent average prediction based on linear mixed-effects analysis, and *thinner grey lines* represent individual patient data

The mean (SD) overall CFD score at treatment initiation in the whole patient cohort was 8.53 (1.81). Figure 1 shows individual and average predicted CFD scores over the entire observation period. In the continued subgroup, mean (SD) CFD scores among 12 patients with available data were 8.42 (1.68) at treatment initiation and 8.67 (1.42) at last follow up, compared with 7.75 (3.30) and 10.75 (4.50) among patients with available data in the discontinued subgroup.

The mean (SD) pre-treatment annual rate of progression of CFD scores in the continued subgroup was 0.75 (0.94) based on patients with both pre- and post miglustat initiation data (*n* = 10), which decreased to 0.29 (1.29) among 12 evaluable patients during the post-baseline period. Mean (SD) annual pre-treatment and post-initiation progression rates among the four evaluable patients in the discontinued subgroup were 0.48 (0.44) and 1.49 (1.31), respectively.

From the linear mixed modelling analysis, the calculated mean (95 % CI) difference in pre-treatment and post-baseline annual CFD progression rates in the continued subgroup was −0.35 (−0.52, −0.18)—a statistically significant reduction (*p* < 0.001). Further, the post-baseline progression rate in this subgroup [0.087 (−0.05, 0.22)] was not statistically significantly different from zero (*p* = 0.193). In the discontinued subgroup, the mean (95 % CI) pre-treatment versus post-baseline difference in annual progression rate [0.82 (0.52, 1.13)] was also statistically significant (*p* < 0.001), indicating an increase in the progression rate. In this subgroup the mean post-baseline progression rate was statistically significantly different compared to zero [1.11 (0.84, 1.38); *p* < 0.001].

### Change in Cho/NAA ratio over time

All patients in the cohort had at least one brain white matter MRS assessment. Mean (SD; range) MRS follow up periods were 2.3 (1.7, 0.5–5.4) years in the overall patient cohort (*n* = 14 evaluable patients), 2.6 (1.9, 0.5–5.4) years in the continued subgroup (*n* = 11 evaluable patients), and 1.4 (0.1, 1.4–1.5) years in the discontinued subgroup (*n* = 3 evaluable patients).

Absolute Cho/NAA ratio values tended to decrease over time during miglustat treatment in the overall patient cohort (Table 3). The mean (SD) Cho/NAA ratio at miglustat initiation was 0.63 (0.12), which decreased to 0.56 (0.09) by 18 (±2) months in the ‘post-baseline’ period. Respective mean (SD) baseline (miglustat initiation) and 18-month values were 0.64 (0.12), and 0.54 (0.07) in the continued subgroup, and 0.57 (0.15) and 0.60 (0.13) in the discontinued subgroup. These data can be considered against mean (SD) normal Cho/NAA ratio values [0.48 (0.076)] from a previous study involving 12 healthy adults (Personal Communication, D Galanaud, 2014). During further long-term follow-up in the continued patient subgroup, albeit based on relatively limited observation numbers, Cho/NAA ratio values were maintained at approximately the same level up to last follow up [mean (SD) 0.43 (0.07) at month 50 ± 2 months; *n* = 3]. One patient from the discontinued subgroup (patient 13) had Cho/NAA ratio values of 0.61 and 0.65 at months 36 and 50, respectively.

**Table 3:** Absolute Cho/NAA ratio values in the centrum semi ovale over time of patients with NP-C

Time point	Whole cohort (*N* = 16)	Continued treatment subgroup (*N* = 12)	Discontinued treatment subgroup (*N* = 4)
Month 0 (baseline), *n* ^a^	14	11	3
Mean (SD)	0.63 (0.12)	0.64 (0.12)	0.57 (0.15)
Median (range)	0.62 (0.46–0.95)	0.64 (0.47–0.95)	0.51 (0.46–0.73)
Month 12, *n* ^a^	9	8	1
Mean (SD)	0.55 (0.08)	0.56 (0.08)	0.50 (NA)
Median (range)	0.55 (0.40–0.68)	0.55 (0.40–0.68)	0.50 (NA)
Month 18, *n* ^a^	8	5	3
Mean (SD)	0.56 (0.09)	0.54 (0.07)	0.60 (0.13)
Median (range)	0.56 (0.44–0.75)	0.57 (0.44–0.60)	0.56 (0.49–0.75)
Month 24, *n* ^a^	5	5	0
Mean (SD)	0.57 (0.26)	0.57 (0.26)	NA
Median (range)	0.52 (0.36–1.02)	0.52 (0.36–1.02)	NA
Month 36, *n* ^a^	4	3	1
Mean (SD)	0.56 (0.09)	0.54 (0.11)	0.61 (NA)
Median (range)	0.57 (0.44–0.65)	0.52 (0.44–0.65)	0.61 (NA)
Month 50, *n* ^a^	4	3	1
Mean (SD)	0.49 (0.12)	0.43 (0.07)	0.65 (NA)
Median (range)	0.47 (0.36–0.65)	0.45 (0.36–0.50)	0.65 (NA)
Month 72, *n* ^a^	1	1	0
Mean (SD)	0.53 (NA)	0.53 (NA)	NA
Median (range)	0.53 (NA)	0.53 (NA)	NA

All measurements were within 2 months of the stated time point

^a^ *n* = number of patients with measurements per time point

**Fig. 2:**
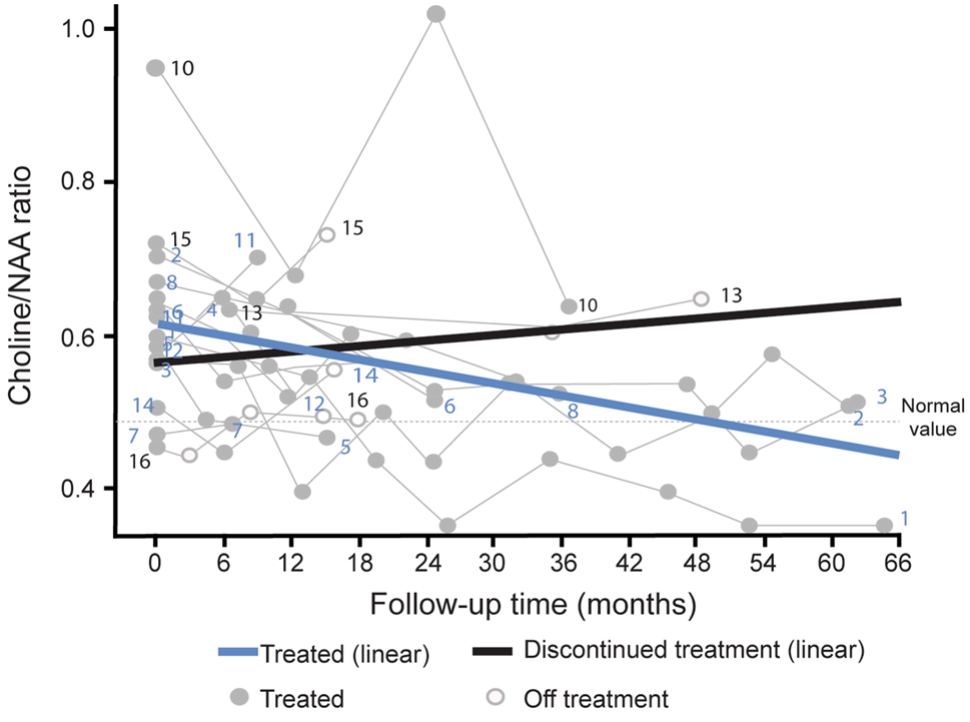
Progression of Cho/NAA ratios over time. *Zero time point* represents the start of follow-up period; *thick coloured lines* represent average prediction based on linear mixed modelling analysis, and *thinner grey lines* represent individual patient profiles. *Dashed horizontal line* represents normal values based on values from healthy controls (Personal Communication, D Galanaud, 2014)

Based on linear mixed modelling analysis (Fig. 2), the mean (95 % CI) annual progression rate of Cho/NAA ratios decreased among patients with available data (*n* = 11) in the continued subgroup by −0.032 (−0.036, −0.027) and increased among three evaluable patients in the discontinued subgroup by 0.015 (–0.005, 0.026).

### Relationship between CFD score and Cho/NAA ratio progression rates

**Fig. 3:**
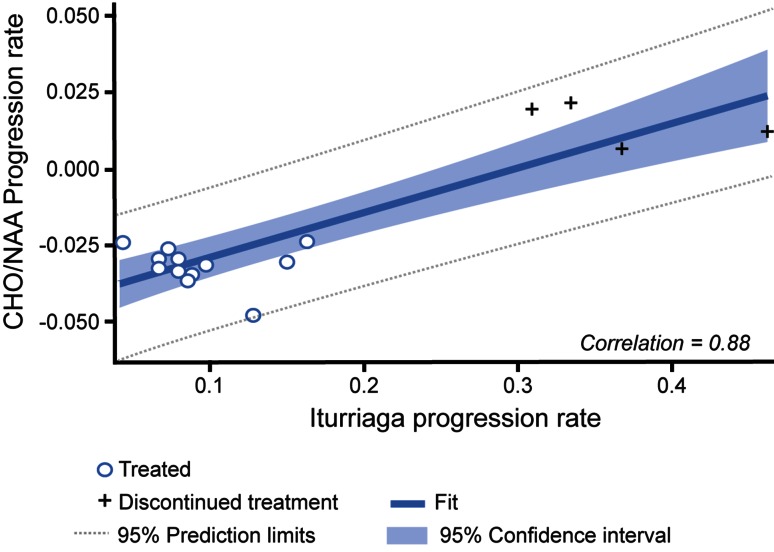
Annual progression rates for CFD score versus Cho/NAA ratio. Data from linear mixed-effects analysis. The *thick central line* is linear regression best-fit line. *Circles* are data from continued patients and crosses are data from discontinued patients

The relationship between CFD and Cho/NAA ratio progression rates was evaluated based on findings from linear mixed regression modelling, with 95 % prediction and confidence intervals (Fig. 3). This analysis showed a high degree of correlation between annual rates of progression for CFD score and annual rates of progression of Cho/NAA ratios, with a correlation coefficient of 0.88 (*p* < 0.001).

## Discussion

In a previous, preliminary study we observed long-term longitudinal changes and normalisation of choline/creatine ratios in three NP-C patients during miglustat therapy, as measured using H-MRS. This indicated the possibility that such H-MRS parameters may be useful as non-invasive surrogate markers of disease progression and treatment efficacy in this disease [8]. In this follow-up, observational study we have extended our initial findings, this time based on the Cho/NAA ratio, in a larger series of 16 adult miglustat-treated NP-C patients over a period of up to 8.7 years. Similar to our previous observation of decreased choline/creatine ratios over 24 months in miglustat-treated patients, the current data indicate sustained reductions in Cho/NAA ratio during long-term, continued miglustat therapy. Baseline mean (SD) Cho/NAA ratios were higher than control values observed during a separate exploratory study in 12 healthy volunteers in our laboratory (Galanaud D, personal communication, 2014), but average values decreased to control levels by month 24 of treatment. This occurred in parallel with stabilisation of clinical disease manifestations (as indicated by CFD scores), which again is in line with previous data [23]. In addition, and perhaps most importantly, changes in the Cho/NAA ratio appeared to correlate with the rate of clinical disease progression.

A major problem in previous trials assessing miglustat in NP-C has been the lack of suitable objective markers that could serve as efficacy endpoints. Neurological examination can be objectified using a number of neurological scales. However, such scales are not fully appropriate for trials in diseases where only small cohorts of patients can be observed, and where the goal is to stabilise neurological manifestations rather than improve neurological lesions that have already occurred. A study conducted by Bowman et al. [3] provided preliminary evidence that brain volumetric measurements may be of use as a more objective and quantitative measure of disease progression in NP-C, but further studies are required to fully evaluate the utility of this approach.

Besides allowing observation of longitudinal changes in Cho/NAA ratio, our findings suggest that H-MRS data may also assist clinical decisions on an individual patient basis. For instance, only three patients (14, 7 and 16) had normal Cho/NAA values before treatment, among whom two (14 and 16) had to stop treatment after 6 and 7 months, respectively, due to adverse effects that overcame any clinical benefit. It is noteworthy that these patients had relatively low rates of disease progression before treatment, which suggests that together with seemingly normal brain function, they did not have very active disease at the time of treatment initiation. We may hypothesise that treatment with miglustat is more beneficial in patients with high baseline Cho/NAA values (indicating more active disease), but is less so in slowly progressing patients. Miglustat therapy might therefore best be introduced cautiously in patients with normal Cho/NAA ratio. Conversely, patients 13 and 15, who both discontinued treatment after 14 months due to perceived lack of efficacy, had very high Cho/NAA ratios (>0.61) after 6–12 months of treatment, which suggests that lack of improvement in Cho/NAA values might be predictive of low treatment response (e.g., based on CFD progression).

Overall, these findings need to be confirmed in a larger patient cohort, as some patients in the current study who showed good clinical response to treatment (e.g., patient 4) also had a high Cho/NAA value even after 12 months. Nevertheless, a working hypothesis could be that the higher the Cho/NAA ratio, the better the expected treatment effect with miglustat, but that above a certain threshold, a highly active disease process might preclude beneficial drug effects.

It is interesting to note that miglustat treatment had to be continued for a prolonged period (approximately 24 months) in order to achieve normalisation and stabilisation of the mean Cho/NAA ratio. This long-term effect is consistent with the mechanism of action of miglustat, which slows down ganglioside synthesis but does not increase the turnover of already accumulated lipids. The half-life of lipids in cell membranes can be very long; that of cholesterol has been estimated to range in the years in humans [2]. Thus, our data, albeit based on a small cohort, suggest that the effect of miglustat on brain lipids is quite slow.

This study has several limitations. Firstly, we lacked a well-matched control untreated patient group for comparison. The data indicate that clinical neurological impairment (based on CFD score) increases with time, and that this impairment decreased during miglustat treatment in parallel with improvements in the Cho/NAA ratio. However, we were only able to compare this treatment effect with estimated pre-treatment data, which was limited to only a few observations in the four patients in the early discontinuation group.

Overall, this study shows reductions in nervous system dysfunction in adults with NP-C during miglustat therapy. We suggest that H-MRS could be used both as a marker for the follow-up as well as a predictor of treatment effectiveness.

## Electronic supplementary material

Below is the link to the electronic supplementary material.

Supplementary material 1 (DOCX 30 kb)
