# Supplementary material for: Normalisation of brain spectroscopy findings in Niemann–Pick disease type C patients treated with miglustat
Source: J Neurol. 2016 Mar 16;263:927–36. doi: 10.1007/s00415-016-8051-1 (PMC4859844; doi:10.1007/s00415-016-8051-1)
Supplement: Supplementary file 1 — Supplementary material 1 (DOCX 30 kb) [file 415_2016_8051_MOESM1_ESM.docx]

**Supplement 1**

**Model 1.** Applied for each patient to estimate their individual progression rate:

y = α + β(t)^-^ + φ(*t*)^+^

*Where the function ‘(t)^+^’ resolves to t if t is positive, and ‘0’ otherwise; and ‘(t)^-^’ resolves to t if t is negative, and ‘0’ otherwise.*

**Model 2.** Analysis of the annual CFD progression rate before miglustat and after start of miglustat therapy by group:

y = α + δx + β(*t*)^-^ + λx(t)^-^ + φ(t)^+^ + yx(*t*)^+^

*The variable ‘x’ represents the patient group (‘1’ for patients who continued treatment and ‘0’ for patients who discontinued treatment); and ‘t’ was as defined for model 1. The variables ‘x’ and ‘t’ are fixed factors, and ‘α’ is a random intercept.*

**Model 3.** Analysis of the annual *Cho/NAA ratio* progression rate before miglustat and after start of miglustat therapy by group:

y = α + βt + αx+ λtx

*Variables ‘x’ and ‘t’ as defined in model 1. The factor ‘x’ is a fixed effect, t’ is a random effect, and ‘α’ is a random intercept.*
